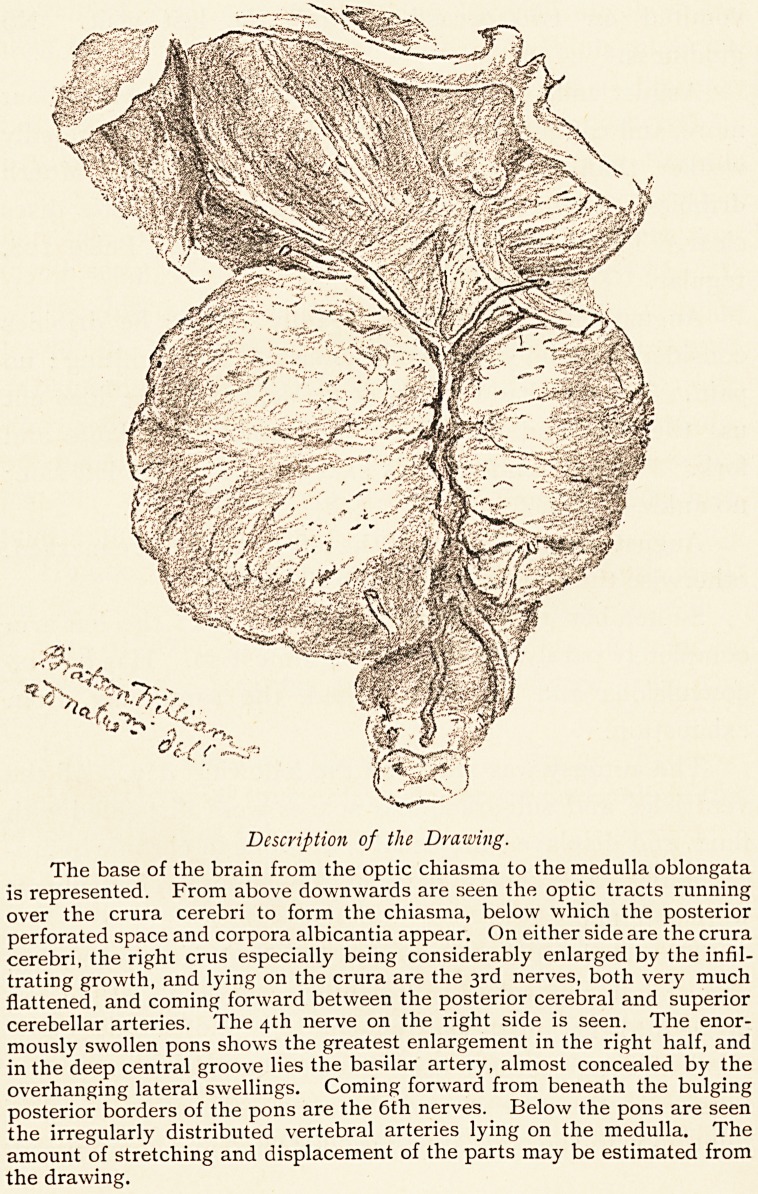# Case of Tumour of the Pons

**Published:** 1891-09

**Authors:** P. Watson Williams

**Affiliations:** Assistant-Physician to the Bristol Royal Infirmary


### CASE OF TUMOUR OF THE PONS.

BY

P. WATSON WILLIAMS, M.B. Lond.,

*Assistant-Physician to the Bristol Royal Infirmary.*

---

ON July 14th, 1890, Reginald P., æt. six, was taken to Dr. Shingleton Smith, on whose behalf I subsequently attended the patient, and who has kindly allowed me to publish the following brief history of the case.

In the Easter holidays of 1889, R. P. was noticed to be tiresome and fretful; but he went back to school at Whitchurch, and there was no complaint of him whilst there, excepting that he had been observed to tumble. He came home for the holidays at the end of June, and he seemed lively and well, but he tumbled about, stumbling at nothing and falling forwards. At Whitsuntide he drove out with his uncle, and he then got out of the trap and ran about, but whilst doing so he was observed to turn round like a top and fall down. He was slightly hydrocephalic.

There was no evidence of syphilis or tubercle, nor any history of convulsions. When first seen by Dr. Smith, his condition was as follows: Face a little drawn to the right: strabismus, the right external rectus being weak: pupils large and inactive: left optic disc blurred: tongue protruded straight: gait unsteady, but no dragging of either leg: patellar tendon-reflex normal: upper limbs not markedly affected, the grasp good and equal.

He does not complain of headache, sleeps fairly well, but is restless, and "can't keep the clothes on him." Is very irritable, and subject to violent fits of temper. No vomiting. Hearing good; no discharge from the ears.

Urine: no albumen, trace of sugar. Sp. gr. 1030.

An intracranial tumour was diagnosed, probably in the neighbourhood of the pons.

July 20th. The symptoms have become more marked; he is still able to walk, but is very unsteady, and the left leg drags at times. Chorea-like jactitation of face muscles, he never keeps still. He has no pain anywhere, sleeps fairly well, appetite good, no vomiting. Pupils large and inactive, but there is no defect of vision or of hearing. Right external rectus weak.

July 26th. The right facial muscles weak and twitching. Strabismus more marked; the right sixth nerve is paralysed. He sleeps better, but is very irritable. Has vomited on two occasions since the last visit. No giddiness.

August 2nd. The patient gets weaker and falls about more, and cannot get up alone. His left arm is decidedly weaker than the right, but is not paralysed. Mouth dribbles. Right external rectus paralysed. Optic discs clear. There has been no more vomiting. Pulse 108, regular. Temperature normal.

August 18th. Is getting steadily worse; he dribbles considerably, and yawns continuously. No vomiting; no pain. The left-hand grasp is feeble, in fact he does not use the left hand at all now; he cannot use a knife and fork. Patellar tendon-reflex exaggerated on the left side; no ankle-clonus.

August 22nd. Paresis of the left hand is more marked, otherwise the condition is much the same.

September 16th. Patient is very weak; the left arm completely paralysed, the left leg almost so. Has had no convulsions and no pain. Died, the same day, from exhaustion.

The autopsy was made on the following day. All the ventricles and sub-arachnoid spaces were distended with fluid, and displayed the usual conditions in chronic hydrocephalus. The central parts of the cerebral hemispheres were softened.

The whole of the pons was involved in new growth, and was much swollen on its anterior surface, and bulging above into the fourth ventricle.

Both crura cerebri were likewise enlarged by the extension of the growth, and the structures and nerves in relation with the pons and crura distorted and compressed, as shown in the drawing, which gives the actual size of the parts represented.

*Description of the Drawing.*

The base of the brain from the optic chiasma to the medulla oblongata is represented. From above downwards are seen the optic tracts running over the crura cerebri to form the chiasma, below which the posterior perforated space and corpora albicantia appear. On either side are the crura cerebri, the right crus especially being considerably enlarged by the infiltrating growth, and lying on the crura are the 3rd nerves, both very much flattened, and coming forward between the posterior cerebral and superior cerebellar arteries. The 4th nerve on the right side is seen. The enormously swollen pons shows the greatest enlargement in the right half, and in the deep central groove lies the basilar artery, almost concealed by the overhanging lateral swellings. Coming forward from beneath the bulging posterior borders of the pons are the 6th nerves. Below the pons are seen the irregularly distributed vertebral arteries lying on the medulla. The amount of stretching and displacement of the parts may be estimated from the drawing.

The third nerves of both sides are seen flattened and displaced. The sixth nerves, too, are winding round the bulging posterior border of the tumour, especially that on the right side, which explains the paralytic condition of the right external rectus muscle.

Externally this infiltrating growth was colourless and almost translucent on the surface. Internally it was red and very vascular.

Microscopical examination showed that it consisted of small round and oval cells enclosed in a granular, finely fibrillated inter-cellular substance, a typical glioma.

This case, like the majority of gliomatous tumours of the pons, shows how impossible it is to determine the extent of the growth before death; for though the symptoms may enable one to roughly locate the tumour, the *post mortem* examination generally reveals a great deal more than the physical signs observed during life would lead one to suspect.

## Figures and Tables

**Figure f1:**